# (−)-(1*S*,5*R*)-2-Oxabicyclo­[3.3.1]nonan-3-one

**DOI:** 10.1107/S1600536810014339

**Published:** 2010-04-24

**Authors:** Teresa Olejniczak, Agata Białońska

**Affiliations:** a Department of Chemistry, Wrocław University of Environmental and Life Sciences, 25. Norwida, 50-375 Wrocław, Poland; b Faculty of Chemistry, University of Wrocław, 14. F. Joliot-Curie, 50-383 Wrocław, Poland

## Abstract

In the title compound, C_8_H_12_O_2_, the cyclo­hexane ring exhibits a chair conformation and the δ-lactone ring is axially bonded to the cyclo­hexane ring. In the crystal, mol­ecules are linked by C—H⋯O hydrogen bonds, resulting in ribbons extending along [010].

## Related literature

For the synthesis and confirmation of the absolute configuration of the title compound, see Olejniczak (2010 ▶); Wascholowski *et al.* (2008 ▶); Tzvetkov *et al.* (2006 ▶); Xu *et al.* (2002 ▶). For related structures see: Yokoyama *et al.* (2003 ▶); Schmidt *et al.* (1998 ▶); Finet *et al.* (2007 ▶); Militsina *et al.* (2005 ▶).
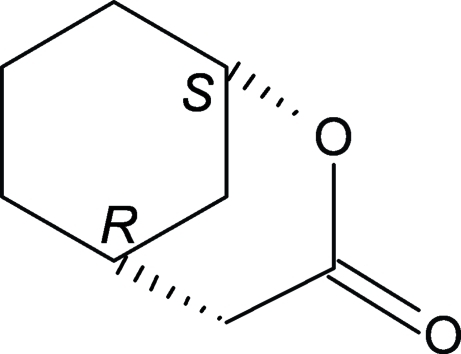


## Experimental

### 

#### Crystal data

C_8_H_12_O_2_
*M* *_r_* = 140.18
Orthorhombic, 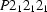

*a* = 6.793 (2) Å
*b* = 7.467 (2) Å
*c* = 14.170 (4) Å
*V* = 718.7 (4) Å^3^
*Z* = 4
Mo *K*α radiation
μ = 0.09 mm^−1^
*T* = 100 K
0.30 × 0.14 × 0.10 mm

#### Data collection

Kuma KM-4-CCD diffractometer
4925 measured reflections
935 independent reflections
685 reflections with *I* > 2σ(*I*)
*R*_int_ = 0.051

#### Refinement

*R*[*F* ^2^ > 2σ(*F* ^2^)] = 0.043
*wR*(*F* ^2^) = 0.096
*S* = 1.04
935 reflections
92 parameters
H-atom parameters constrained
Δρ_max_ = 0.35 e Å^−3^
Δρ_min_ = −0.16 e Å^−3^

### 

Data collection: *CrysAlis CCD* (Oxford Diffraction, 2009 ▶); cell refinement: *CrysAlis RED* (Oxford Diffraction, 2009 ▶); data reduction: *CrysAlis RED*; program(s) used to solve structure: *SHELXS97* (Sheldrick, 2008 ▶); program(s) used to refine structure: *SHELXL97* (Sheldrick, 2008 ▶); molecular graphics: *XP* (Bruker, 1999 ▶); software used to prepare material for publication: *SHELXL97*.

## Supplementary Material

Crystal structure: contains datablocks global, I. DOI: 10.1107/S1600536810014339/hg2667sup1.cif

Structure factors: contains datablocks I. DOI: 10.1107/S1600536810014339/hg2667Isup2.hkl

Additional supplementary materials:  crystallographic information; 3D view; checkCIF report

## Figures and Tables

**Table 1:** Hydrogen-bond geometry (Å, °)

*D*—H⋯*A*	*D*—H	H⋯*A*	*D*⋯*A*	*D*—H⋯*A*
C1—H1⋯O2^i^	1.00	2.58	3.224 (3)	122

Symmetry code: (i) 
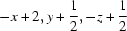
.

**Fig. 1.**
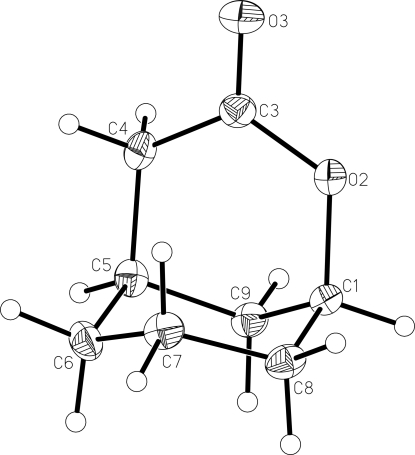
Selected view of (-)-3 (30% probability thermal ellipsoids).

**Fig. 2.**
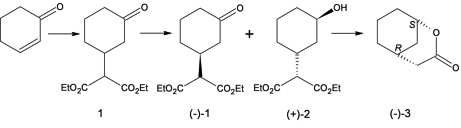
Scheme of a three step synthesis of (-)-3.

**Fig. 3.**
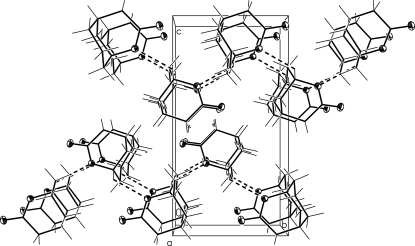
Packing of (-)-3.

## supplementary crystallographic information

### Comment

The titled compound, C_8_H_12_O_2_, was prepared in a three step synthesis (Fig. 2). Racemic diethyl 2-(3-oxocyclohexyl)malonate (1) was synthesized as a product of Michael addition of diethyl malonate to cyclohex-2-en-1-one. (-)-Diethyl 2-((*S*)-3-oxocyclohexyl)malonate ((-)-1) (ee=98%) and (+)-diethyl 2-((1*R*, 3*R*)-3-hydroxycyclohexyl)malonate ((+)-2) (ee=99%) were isolated by micriobial bioreduction using *Absidia coerulea* AM 93. Hydroxydiester-(+)-2 was subjected to chemical lactonization, leading to (-)-(1*S*, 5*R*)-2-oxabicyclo[3.3.1]nonan-3-one ((-)-3) [for more details see Olejniczak, 2010].

The molecular structure of the title compound is shown in Fig. 1. Bond lengths and angles in (-)-3 are similar to those observed in related structures [Yokoyama *et al.*, 2003; Schmidt *et al.*, 1998; Finet *et al.*, 2007]. As in these related structures, in (-)-3 the cyclohexane ring reveals *chair* conformation (Fig. 1) and the δ-lactone ring is axially bonded to the cyclohexane ring.

It is worth mentioning that the conformation of the δ-lactone ring differs a little from those observed in the related structures. According to the numbering scheme employed in this paper, the torsion angles C1 O2 C3 C4 and O2 C3 C4 C5 in the related structures are in the range -7.1 - 0.4 ° and 0.0 - 8.8 °, respectively, and in (-)-3 values of suitable torsion angles are equal to -18.1 (3) and 23.3 (4) °. However, the values of the torsion angles are similar to those, -17.5 and 25.5 °, observed in one of crystallographically unrelated molecules of 3,9,12a-trimethyl-5-oxotetradecahydro-3,6a- methanonaphtho[2,1-d]oxocine-9-carboxylic acid, in which δ-lactone ring axially bonded to cyclohexane ring is observed [Militsina *et al.*, 2005].

The structure of (-)-3 is stabilized by weak intermolecular C—H···O hydrogen bonds and van der Waals contacts. Molecules of (-)-3 are linked by the C1—H1···O2(2-x, 0.5+y, 0.5-z) hydrogen bonds, resulting in ribbons extended along the [010] direction (Table 1, Fig. 3).

### Experimental

Crystals suitable for X-ray structure analysis were obtained directly after purification by column chromatography by slow evaporation of the eluent (petroleum ether : aceton : *iso*-propanol : ethyl acetate (40:1:3:1) v/v) at room temperature.

### Refinement

All H atoms were placed at calculated positions and were treated as riding atoms, with C—H distances of 0.99 - 1.00 Å. The absolute configuration of (-)-3 was choosen on the basis of known absolute configuration of particular substrates: The absolute configuration of (-)-1 was confirmed by comparison of its optical rotation with the literature data [Wascholowski *et al.*, 2008; Tzvetkov *et al.*, 2006; Xu *et al.*, 2002]. The absolute configuration of the carbon atom bearing hydroxyl group in product (+)-2 was determined using the Mosher's ester [Olejniczak, 2010].

### Crystal data

**Table:**

C_8_H_12_O_2_	*F*(000) = 304
*M**_r_* = 140.18	*D*_x_ = 1.295 Mg m^−^^3^
Orthorhombic, *P*2_1_2_1_2_1_	Mo *K*α radiation, λ = 0.71073 Å
Hall symbol: P 2ac 2ab	Cell parameters from 1754 reflections
*a* = 6.793 (2) Å	θ = 3.0–28.8°
*b* = 7.467 (2) Å	µ = 0.09 mm^−^^1^
*c* = 14.170 (4) Å	*T* = 100 K
*V* = 718.7 (4) Å^3^	Needle, colorless
*Z* = 4	0.30 × 0.14 × 0.10 mm

### Data collection

**Table:**

Kuma KM-4-CCD diffractometer	685 reflections with *I* > 2σ(*I*)
Radiation source: fine-focus sealed tube	*R*_int_ = 0.051
graphite	θ_max_ = 27.0°, θ_min_ = 3.1°
ω scan	*h* = −8→8
4925 measured reflections	*k* = −9→9
935 independent reflections	*l* = −15→18

### Refinement

**Table:**

Refinement on *F*^2^	Primary atom site location: structure-invariant direct methods
Least-squares matrix: full	Secondary atom site location: difference Fourier map
*R*[*F*^2^ > 2σ(*F*^2^)] = 0.043	Hydrogen site location: inferred from neighbouring sites
*wR*(*F*^2^) = 0.096	H-atom parameters constrained
*S* = 1.04	*w* = 1/[σ^2^(*F*_o_^2^) + (0.0507*P*)^2^] where *P* = (*F*_o_^2^ + 2*F*_c_^2^)/3
935 reflections	(Δ/σ)_max_ = 0.004
92 parameters	Δρ_max_ = 0.35 e Å^−^^3^
0 restraints	Δρ_min_ = −0.16 e Å^−^^3^

### Special details

**Table:**

Geometry. All esds (except the esd in the dihedral angle between two l.s. planes) are estimated using the full covariance matrix. The cell esds are taken into account individually in the estimation of esds in distances, angles and torsion angles; correlations between esds in cell parameters are only used when they are defined by crystal symmetry. An approximate (isotropic) treatment of cell esds is used for estimating esds involving l.s. planes.
Refinement. Refinement of *F*^2^ against ALL reflections. The weighted *R*-factor wR and goodness of fit *S* are based on *F*^2^, conventional *R*-factors *R* are based on *F*, with *F* set to zero for negative *F*^2^. The threshold expression of *F*^2^ > σ(*F*^2^) is used only for calculating *R*-factors(gt) etc. and is not relevant to the choice of reflections for refinement. *R*-factors based on *F*^2^ are statistically about twice as large as those based on *F*, and *R*- factors based on ALL data will be even larger.

### Fractional atomic coordinates and isotropic or equivalent isotropic displacement parameters (Å^2^)

**Table:**

	*x*	*y*	*z*	*U*_iso_*/*U*_eq_	
C1	0.9078 (4)	0.4787 (3)	0.29810 (17)	0.0293 (6)	
H1	1.0256	0.4916	0.2564	0.035*	
O2	0.8893 (3)	0.2882 (2)	0.32719 (12)	0.0331 (5)	
C3	0.7920 (4)	0.2414 (4)	0.40668 (17)	0.0302 (6)	
O3	0.7539 (3)	0.0838 (2)	0.41770 (13)	0.0389 (5)	
C4	0.7539 (4)	0.3815 (3)	0.48057 (18)	0.0300 (6)	
H4A	0.8553	0.3693	0.5304	0.036*	
H4B	0.6247	0.3561	0.5100	0.036*	
C5	0.7535 (4)	0.5754 (3)	0.44672 (17)	0.0287 (6)	
H5	0.7674	0.6561	0.5027	0.034*	
C6	0.5660 (4)	0.6256 (4)	0.39356 (19)	0.0347 (7)	
H6B	0.4509	0.6041	0.4349	0.042*	
H6A	0.5695	0.7549	0.3782	0.042*	
C7	0.5413 (4)	0.5192 (4)	0.30310 (17)	0.0344 (7)	
H7B	0.4290	0.5683	0.2669	0.041*	
H7A	0.5108	0.3931	0.3189	0.041*	
C8	0.7258 (4)	0.5254 (4)	0.24230 (17)	0.0334 (6)	
H8B	0.7115	0.4402	0.1892	0.040*	
H8A	0.7407	0.6471	0.2155	0.040*	
C9	0.9334 (4)	0.5985 (4)	0.38359 (19)	0.0302 (7)	
H9B	0.9457	0.7250	0.3635	0.036*	
H9A	1.0542	0.5648	0.4184	0.036*	

### Atomic displacement parameters (Å^2^)

**Table:**

	*U*^11^	*U*^22^	*U*^33^	*U*^12^	*U*^13^	*U*^23^
C1	0.0333 (15)	0.0256 (14)	0.0290 (14)	0.0004 (12)	0.0060 (13)	0.0044 (12)
O2	0.0360 (11)	0.0311 (10)	0.0322 (10)	0.0031 (8)	0.0082 (9)	−0.0003 (9)
C3	0.0285 (15)	0.0334 (16)	0.0286 (14)	0.0022 (12)	−0.0040 (12)	0.0043 (12)
O3	0.0434 (11)	0.0284 (10)	0.0451 (11)	−0.0025 (10)	−0.0047 (11)	0.0059 (9)
C4	0.0245 (14)	0.0393 (15)	0.0261 (12)	−0.0021 (14)	0.0014 (13)	0.0003 (11)
C5	0.0291 (14)	0.0297 (14)	0.0273 (13)	−0.0034 (13)	−0.0009 (13)	−0.0068 (11)
C6	0.0268 (15)	0.0336 (16)	0.0435 (17)	0.0048 (12)	0.0036 (13)	−0.0038 (13)
C7	0.0284 (14)	0.0368 (16)	0.0381 (17)	−0.0040 (12)	−0.0047 (12)	0.0040 (14)
C8	0.0429 (17)	0.0314 (14)	0.0259 (13)	−0.0055 (14)	−0.0033 (13)	0.0012 (11)
C9	0.0242 (14)	0.0312 (15)	0.0353 (16)	−0.0039 (12)	−0.0012 (12)	0.0037 (12)

### Geometric parameters (Å, °)

**Table:**

C1—O2	1.486 (3)	C5—H5	1.0000
C1—C8	1.509 (3)	C6—C7	1.517 (4)
C1—C9	1.516 (4)	C6—H6B	0.9900
C1—H1	1.0000	C6—H6A	0.9900
O2—C3	1.352 (3)	C7—C8	1.522 (3)
C3—O3	1.215 (3)	C7—H7B	0.9900
C3—C4	1.502 (4)	C7—H7A	0.9900
C4—C5	1.526 (3)	C8—H8B	0.9900
C4—H4A	0.9900	C8—H8A	0.9900
C4—H4B	0.9900	C9—H9B	0.9900
C5—C9	1.524 (3)	C9—H9A	0.9900
C5—C6	1.526 (4)		
			
O2—C1—C8	107.3 (2)	C7—C6—H6B	109.1
O2—C1—C9	110.7 (2)	C5—C6—H6B	109.1
C8—C1—C9	112.1 (2)	C7—C6—H6A	109.1
O2—C1—H1	108.9	C5—C6—H6A	109.1
C8—C1—H1	108.9	H6B—C6—H6A	107.9
C9—C1—H1	108.9	C6—C7—C8	111.8 (2)
C3—O2—C1	121.32 (19)	C6—C7—H7B	109.3
O3—C3—O2	117.5 (2)	C8—C7—H7B	109.3
O3—C3—C4	123.2 (2)	C6—C7—H7A	109.3
O2—C3—C4	119.0 (2)	C8—C7—H7A	109.3
C3—C4—C5	116.2 (2)	H7B—C7—H7A	107.9
C3—C4—H4A	108.2	C1—C8—C7	111.8 (2)
C5—C4—H4A	108.2	C1—C8—H8B	109.3
C3—C4—H4B	108.2	C7—C8—H8B	109.3
C5—C4—H4B	108.2	C1—C8—H8A	109.3
H4A—C4—H4B	107.4	C7—C8—H8A	109.3
C9—C5—C4	106.9 (2)	H8B—C8—H8A	107.9
C9—C5—C6	110.59 (19)	C1—C9—C5	108.1 (2)
C4—C5—C6	112.9 (2)	C1—C9—H9B	110.1
C9—C5—H5	108.8	C5—C9—H9B	110.1
C4—C5—H5	108.8	C1—C9—H9A	110.1
C6—C5—H5	108.8	C5—C9—H9A	110.1
C7—C6—C5	112.4 (2)	H9B—C9—H9A	108.4
			
C8—C1—O2—C3	−85.9 (3)	C4—C5—C6—C7	−63.5 (3)
C9—C1—O2—C3	36.8 (3)	C5—C6—C7—C8	−50.9 (3)
C1—O2—C3—O3	167.8 (2)	O2—C1—C8—C7	65.6 (3)
C1—O2—C3—C4	−18.1 (3)	C9—C1—C8—C7	−56.1 (3)
O3—C3—C4—C5	−162.9 (3)	C6—C7—C8—C1	50.3 (3)
O2—C3—C4—C5	23.3 (4)	O2—C1—C9—C5	−59.8 (3)
C3—C4—C5—C9	−46.0 (3)	C8—C1—C9—C5	60.0 (3)
C3—C4—C5—C6	75.9 (3)	C4—C5—C9—C1	64.0 (2)
C9—C5—C6—C7	56.2 (3)	C6—C5—C9—C1	−59.3 (3)

### Hydrogen-bond geometry (Å, °)

**Table:**

*D*—H···*A*	*D*—H	H···*A*	*D*···*A*	*D*—H···*A*
C1—H1···O2^i^	1.00	2.58	3.224 (3)	122

Symmetry codes: (i) −*x*+2, *y*+1/2, −*z*+1/2.
